# Variational Bayesian causal connectivity analysis for fMRI

**DOI:** 10.3389/fninf.2014.00045

**Published:** 2014-05-05

**Authors:** Martin Luessi, S. Derin Babacan, Rafael Molina, James R. Booth, Aggelos K. Katsaggelos

**Affiliations:** ^1^Athinoula A. Martinos Center for Biomedical Imaging, Harvard Medical School, Massachusetts General HospitalCharlestown, MA, USA; ^2^Department of Electrical Engineering and Computer Science, Northwestern UniversityEvanston, IL, USA; ^3^Google Inc.Mountain View, CA, USA; ^4^Departamento de Ciencias de la Computación e I.A., Universidad de GranadaGranada, Spain; ^5^Department of Communication Sciences and Disorders, Northwestern UniversityEvanston, IL, USA

**Keywords:** fMRI, causality, connectivity, variational Bayesian method, Granger causality

## Abstract

The ability to accurately estimate effective connectivity among brain regions from neuroimaging data could help answering many open questions in neuroscience. We propose a method which uses causality to obtain a measure of effective connectivity from fMRI data. The method uses a vector autoregressive model for the latent variables describing neuronal activity in combination with a linear observation model based on a convolution with a hemodynamic response function. Due to the employed modeling, it is possible to efficiently estimate all latent variables of the model using a variational Bayesian inference algorithm. The computational efficiency of the method enables us to apply it to large scale problems with high sampling rates and several hundred regions of interest. We use a comprehensive empirical evaluation with synthetic and real fMRI data to evaluate the performance of our method under various conditions.

## 1. Introduction

Traditionally, functional neuroimaging has been used to obtain spatial maps of brain activation, e.g., using functional magnetic resonance imaging (fMRI) or positron emission tomography (PET), or to study the spatio-temporal progression of activity using magneto- or electroencephalography (M/EEG). Due to the increasing availability of MRI scanners to researchers and due to their high spatial resolution, the question of how fMRI can be used to obtain measures of *effective connectivity*, describing directed influence and causality in brain networks (Friston, 1994), has recently received significant attention.

An idea that forms the basis of several methods is that causality can be used to infer effective connectivity, i.e., if activity in one region can be used to accurately predict future activity in another region, it is likely that a directed connection between the regions exists. An exhaustive review of causality based methods for fMRI is beyond the scope of this work; we only provide a short introduction and refer to Roebroeck et al. (2011) for a recent review of related methods. Effective connectivity methods for fMRI can be divided into two groups. Methods in the first group are referred to as *dynamic causal modeling* (DCM) methods (Friston et al., 2003). In DCM, the relationship between neuronal activity in different regions of interest (ROIs) is described by bilinear ordinary differential equations (ODEs) and the fMRI observation process is modeled by a biophysical model based on the Balloon model (Buxton et al., 1998, 2004). While providing an accurate model of the hemodynamic process underlying fMRI, the non-linearity of the observation model poses difficulties when estimating the latent variables describing the neuronal activity from the fMRI observations. Due to this, DCM is typically used for small numbers of ROIs (less than 10) and DCM methods typically are confirmatory approaches, i.e., the user provides a number of different candidate models describing the connectivity, which are then ranked based on an approximation to the model evidence.

The second class of methods attempts to estimate effective connectivity between ROIs from causal interactions that exist in the observed fMRI time series. In the widely used *Wiener–Granger causality* (WGC) measure (Wiener, 1956; Granger, 1969) (refer to Bressler and Seth, 2010 for a recent review of related methods), a linear prediction model is employed to predict the future of one time series using either only its past or its past and the past of the time series from a different ROI. If the latter leads to a significantly lower prediction error, the other time series is considered to exert a causal influence on the time series being evaluated, which is indicative of directed connectivity between the underlying ROIs. Related methods estimate the causal connectivity between all time series simultaneously by employing a vector autoregressive (VAR) model. The magnitudes of the estimated VAR coefficients are considered a measure of connectivity between regions. In Valdés-Sosa et al. (2005), a first order VAR model is employed and the connectivity graph is assumed to be sparse, i.e., only few regions are connected. The sparsity assumption is formalized by using ℓ_1_-norm regularization of the VAR coefficients. It has been shown in Haufe et al. (2008) that the use of higher order VAR models in combination with ℓ_1_ℓ_2_-norm (group-lasso) (Yuan and Lin, 2006; Meier et al., 2008) regularization of the VAR coefficients across lags leads to a more accurate estimation of the connectivity structure.

There are two main concerns when estimating effective connectivity from causal relations in the observed fMRI time series. First, the processing times at the neuronal level are in the order of milliseconds, which is several orders of magnitude shorter than the sampling interval (time to repeat, TR) of the MRI scanner. Second, fMRI measures neuronal activity indirectly through the so-called blood oxygen level dependent (BOLD) contrast (Ogawa et al., 1990; Frahm et al., 1992), which depends on slow hemodynamic processes. The observation process can be modeled as a convolution of the time series describing the neuronal activity with a hemodynamic response function (HRF). As there is variability in the shape of the HRF among brain regions and individuals (Handwerker et al., 2004) and the sampling rate of the MRI scanner is low, detecting effective connectivity from causal interactions that exist in the observed fMRI data is a challenging problem. There has recently been some controversy if this is indeed the case. In David et al. (2008), a study using simultaneous fMRI, EEG, and intra-cerebral EEG recordings from rats was performed and it was found that the performance of WGC for fMRI is indeed poor, unless the fMRI time series of each region is first deconvolved with the measured HRF of the same region. Using simulations with synthetic fMRI data generated using the biophysical model underlying DCM, it was also found in Smith et al. (2011) that WGC methods perform poorly relative to the other evaluated connectivity methods. On the other hand, another recent study (Deshpande et al., 2010) found that WGC methods provide a high accuracy for the detection of causal interactions at the neuronal level with interaction lengths of hundreds of milliseconds, i.e., much shorter than the TR of the MRI scanner, even when HRF variations are present. The minor influence of HRF variations may be explained by the property that typical HRF variations do not simply correspond to temporal shifts of an HRF with the same shape, which would change the causality of interactions present in the fMRI data. Instead, as pointed out in Deshpande et al. (2010), the HRF variability among brain regions is mostly apparent in the shape of the peak of the HRF and the time-to-peak (Handwerker et al., 2004), which may explain why causal interactions at the neuronal level can still be present after convolution with varying HRFs. This is in agreement with recent results. It has been shown that WGC is invariant to filtering with invertible filters (Barnett and Seth, 2011) and in Seth et al. (2013) simulations were performed that confirm that the invariance typically holds for HRF convolution. However, at the same time it was found that WGC can be severely confounded when HRF convolution is combined with downsampling and measurement noise is added to the data.

Several methods have been proposed that account for HRF variability when analyzing WGC from fMRI data. In David et al. (2008) a noise-regularized HRF deconvolution was employed. and in Smith et al. (2010) a switching linear dynamical system (SLDS) model is proposed to describe the interaction between latent variables representing the neuronal activity together with a linear observation model based on a convolution with a (unknown) HRF for each region. The method employs a Bayesian formulation and obtains estimates of the latent variables using the maximum-likelihood approach. In contrast to WGC methods, the SLDS model can also account for modulatory inputs which change the effective connectivity of the network and introduce non-stationarity in the observed fMRI data. The method in Smith et al. (2010) can be seen as a convergence of DCM methods and WGC-type methods (Roebroeck et al., 2011). A similar method is proposed in Ryali et al. (2011), which can be considered a multivariate extension of methods which perform deconvolution of the neuronal activity for a single fMRI time series (Penny et al., 2005; Makni et al., 2008). Joint estimation of the HRF and detection of neuronal activity is also an important problem for event-related fMRI, we refer to Cassidy et al. (2012) and Chaari et al. (2013) for recently proposed methods addressing this problem.

In this paper, we propose a causal connectivity method for fMRI which employs a VAR model of arbitrary order for the time series of neuronal activity in combination with a linear hemodynamic convolution model for the fMRI observation process. We use a Bayesian formulation of the problem and draw inference based on an approximation to the posterior distribution which we obtain using the variational Bayesian (VB) method (Jordan et al., 1999; Attias, 2000). In contrast to previous methods (Smith et al., 2010; Ryali et al., 2011), our method is designed to be computationally efficient, enabling application to large scale problems with large numbers of regions and high temporal sampling rates. Computational efficiency is achieved by the introduction of an approximation to the neuronal time series in the Bayesian modeling. When drawing inference, introducing this approximation has the effect that the hemodynamic deconvolution can be separated from the estimation of the neuronal time series, leading to a reduction of the state-space dimension of the variational Kalman smoother (Beal and Ghahramani, 2001; Ghahramani and Beal, 2001), which forms a part of the VB inference algorithm. The lower state-space dimension drastically reduces the processing and memory requirements. Another key difference to previous Bayesian methods is that we assume that the VAR coefficient matrices are sparse and that the coefficient matrices at different lags have non-zero entries at mostly the same locations, i.e., the matrices have similar sparsity profiles. In Haufe et al. (2008) this assumption is formalized using an ℓ_1_ℓ_2_-norm regularization term for the VAR coefficient matrices. In our work, we employ Gaussian priors with shared precision hyperparameters for the VAR coefficient matrices, which is a Bayesian alternative to ℓ_1_ℓ_2_-norm regularization and results in a higher estimation performance of the method.

Our results show that the proposed method offers a higher detection performance than WGC when the number of nodes is large or when the SNR is low. In addition, our method is less affected when the VAR model order assumed in the method is higher than the order present in the data. We also perform simulations using a modified version of our method, which is similar to the method in Ryali et al. (2011), and show that the approximation to the neuronal time series used in our method has a negligible effect on the estimation performance while allowing the application of the proposed method to large problems with hundreds of ROIs. We perform an extensive series of simulations where we vary both the downsampling ratio and the neuronal delay. The results show that the proposed method offers some benefits over WGC, especially in low SNR situations and when HRF variations are present. However, both the proposed method and WGC can at times detect a causal influence with the opposite direction of the true influence, which is a known problem for WGC methods (David et al., 2008; Deshpande et al., 2010; Seth et al., 2013). Finally, we apply the proposed method to resting-state fMRI data from the Human Connectome Project (Van Essen et al., 2012), where it successfully detects connections between regions that belong to known resting-state networks.

This paper is outlined as follows. First, we introduce a hierarchical Bayesian formulation for the generative model underlying the fMRI connectivity estimation problem. Next, we present the Bayesian inference scheme which estimates the latent variables of the model using a variational approximation to the posterior distribution. We then perform extensive simulations with synthetic fMRI data. Finally, we apply the method to real fMRI data and conclude the paper.

### 1.1. Notation

We use the following notation throughout this work: Matrices are denoted by uppercase bold letters, e.g., **A**, while vectors are denoted by lowercase bold letters, e.g., **a**. The element at the *i*-th row and *j*-th column of matrix **A** is denoted by *a*_*ij*_, while **a**_*i*·_ and **a**_·*j*_ denote column vectors with the elements from the *i*-th row and the *j*-th column of **A**, respectively. The operator diag (**A**) extracts the main diagonal of **A** as a column vector, whereas Diag (**a**) is a diagonal matrix with **a** as its diagonal. The operator vec (**A**) vectorizes **A** by stacking its columns, tr (**A**) denotes the trace of matrix **A**, and ⊗ denotes the Kronecker product. The identity matrix of size *N* × *N* is denoted by **I**_*N*_. Similarly, **0**_*N*_ and **0**_*N* × *M*_ denote *N* × *N* and *N* × *M* all-zero matrices, respectively.

## 2. Bayesian modeling

The goal of this work is to infer effective connectivity implied by the causal relations between *N* time series of neuronal activity from *N* different regions in the brain. To this end, we employ a vector autoregressive (VAR) model of order *P* to model the time series as follows

(1) $$s\left(t\right)=\sum_{p\;=\;1}^{P}A^{\left(p\right)}s\left(t-p\right)+\eta \left(t\right),$$

where **s** (*t*) ∈ ℝ^*N*^ denotes the neuronal activity of all regions at time *t*, **A**^(*p*)^ ∈ ℝ^*N* × *N*^ is a matrix with VAR coefficients for lag *p*, and **η**(*t*) ~ 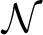
(0, **Λ**^−1^) denotes the innovation. In this model, the activity at any time point is predicted from the activity at *P* previous time points. More specifically, the activity of the *i*-th time series at time *t*, denoted by *s*_*i*_(*t*), is predicted from the past of the *j*-th time series using the coefficients {*a*^(*p*)^_*ij*_}^*P*^_*p* = 1_. Hence, if any of these coefficients is significantly larger than zero, we can conclude that the *j*-th time series exerts a causal influence on the *i*-th time series, implying connectivity between the regions. This is the idea underlying Wiener–Granger causality (Wiener, 1956; Granger, 1969) and related methods using vector autoregressive models (Valdés-Sosa et al., 2005; Haufe et al., 2008).

We can now introduce an embedding process (Weigend and Gershenfeld, 1994; Penny et al., 2005) **x** (*t*) defined by

(2) $$x\left(t\right)={\left[s{\left(t\right)}^{T}s{\left(t-1\right)}^{T}\ldots s{\left(t-P+1\right)}^{T}\right]}^{T},$$

which allows us to express (Equation (1)) by a first order VAR model as follows

(3) $$x\left(t\right)=\tilde{A}x\left(t-1\right)+\tilde{\eta }\left(t\right),$$

where **Ã** ∈ ℝ^*PN* × *PN*^ is given by

(4) $$\tilde{A}=\left[\begin{array}{ccccc} A^{\left(1\right)} & A^{\left(2\right)} & \cdots & A^{\left(P-1\right)} & A^{\left(P\right)}\\ I_{N} & 0_{N} & \cdots & 0_{N} & 0_{N}\\ 0_{N} & I_{N} & \cdots & 0_{N} & 0_{N}\\ \vdots & \vdots & \ddots & \vdots & \vdots \\ 0_{N} & 0_{N} & \cdots & I_{N} & 0_{N} \end{array}\right].$$

The innovation $\tilde{\eta }\left(t\right)$ is Gaussian 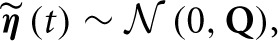, where the covariance matrix **Q** is all zero, except for the first *N* rows and columns, which are given by **Λ**^−1^. For the remainder of this paper, we present the modeling and inference with respect to the time series **x** (*t*). If access to the neuronal time series **s** (*t*) is required, it can easily be extracted from **x** (*t*) (it simply corresponds to the first *N* elements of **x** (*t*)).

### 2.1. Observation model

Before introducing the observation model, note that we can obtain a noisy version of the neuronal time series from the embedding process **x** (*t*) as follows

(5) z(t)=Bx(t)+κ(t),

where **B** = [**I**_*N*_ **0**_*N* × (*P* − 1)*N*_] and **κ** (*t*) ~ 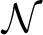
(0, ϑ^−1^**I**), where ϑ is the precision parameter. Clearly, by using very large values for ϑ, the time series **z** (*t*) approaches **s** (*t*). The introduction of this Gaussian approximation to the neuronal time series greatly improves the computational efficiency of the proposed method, as it separates the VAR model for the neuronal time series from the hemodynamic observation model. This separation leads to a reduction of the state-space dimension of the Kalman smoothing algorithm, which forms part of the inference procedure, and therefore to greatly reduced memory requirements. In addition, using the approximation allows us to perform parts of the estimation in the frequency domain, which is computationally advantageous due to the efficiency of the fast Fourier transform. The computational advantages of the proposed method will be discussed in detail in the next section.

To model the fMRI observation process, we follow the standard assumption underlying the general linear model (Friston et al., 1995), and express the fMRI observation of the *i*-th region as follows

(6) $$\begin{array}{l} y_{i}\left(t\right)=h_{i}\left(t\right)*z_{i}\left(t\right)+\varepsilon_{i}\left(t\right)\\ \;=\sum_{k=1}^{L}h_{i}\left(k\right)z_{i}\left(t-k+1\right)+\varepsilon_{i}\left(t\right), \end{array}$$

where * denotes the convolution operation, *h*_*i*_ (*t*) is the hemodynamic response function (HRF) of length *L* for the *i*-th region, and ε_*i*_ (*t*) denotes observation noise. Notice that we can arrange the HRF *h*_*i*_ (*t*) into a *T* × *T* convolution matrix **H**_*i*_, which allows us to write (Equation (6)) as

(7) $$y_{i}=H_{i}z_{i}+\varepsilon_{i},$$

where the *T* × 1 vectors **y**_*i*_, **z**_*i*_, and **ε**_*i*_ are the fMRI observation, the approximation to the neuronal signal, and the observation noise, for the *i*-th region, respectively.

### 2.2. VAR coefficient prior model

We proceed by defining priors for the VAR coefficient matrices {**A**^(*p*)^}^*P*^_*p* = 1_. For a network consisting of a large number of regions, it can generally be assumed that the connectivity is sparse, i.e., the VAR coefficient matrices contain a small number of non-zero coefficients. In the context of inferring causal connectivity, this idea has been used in Valdés-Sosa et al. (2005), where a first order VAR model with ℓ_1_-norm regularization for the VAR coefficients is used to obtain a sparse solution. For higher order VAR models, it is intuitive to assume that if the VAR coefficient *a*^(*p*_1_)^_*ij*_ modeling the connectivity from region *j* to region *i* and lag *p*_1_ is non-zero, it is likely that also other VAR coefficients for the same connection but different lags, i.e., *a*^(*p*_2_)^_*ij*_, *p*_2_ ≠ *p*_1_, are also non-zero. Together with the sparsity assumption, this leads to VAR coefficient matrices with similar sparsity profiles, i.e., the coefficient matrices at different time lags have non-zero entries at mostly the same locations. In Haufe et al. (2008) this idea is formalized by using ℓ_1_ℓ_2_-norm (group lasso) (Yuan and Lin, 2006; Meier et al., 2008) regularization for the VAR coefficients across different lags, resulting in an improved estimation performance in comparison to methods that use alternative forms of regularization, such as, ℓ_1_-norm or ridge regression.

We incorporate the group sparsity assumption using Gaussian priors with shared precision hyperparameters across different lags. More specifically, we use

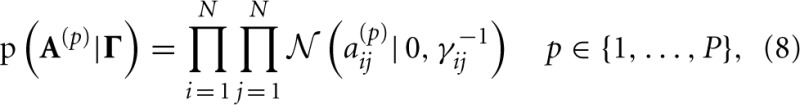



with Jeffreys hyperpriors to the precision hyperparameters

(9) $$\text{p}\left(\Gamma \right)\propto \prod_{i=1}^{N}\prod_{j=1}^{N}{\left(\gamma_{ij}\right)}^{-1}.$$

During estimation, most of the precision hyperparameters in **Γ** will assume very large values, hence effectively forcing the corresponding VAR coefficients to zero. This formulation is an adaptation of sparse Bayesian learning (also known as automatic relevance determination, ARD) (Tipping, 2001) to the problem of VAR coefficient estimation and can be considered a Bayesian alternative to a deterministic ℓ_1_ℓ_2_-norm regularization term. Formulations where shared precision hyperparameters are used to enforce group sparsity have recently been proposed for applications such as simultaneous sparse approximation (Wipf and Rao, 2007), where shared precision parameters are used to obtain solutions with similar sparsity profiles across multiple time points. Recently, shared hyperparameters were used to model the low-rank structure of the latent matrix in matrix estimation (Babacan et al., 2012).

### 2.3. Innovation and noise prior models

To complete the description of the Bayesian model, we define priors for the innovation process and the observation noise in Equations (1) and (6), respectively. We assume that the innovations are independent and identically distributed (i.i.d.) zero-mean Gaussian for each time point, i.e., **η** (*t*) ~ 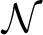
(0, **Λ**^−1^) and **ε** (*t*) ~ 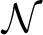
(0, **R**). It has to be expected that the linear prediction model used in the proposed method cannot fully explain the relationship between the neuronal time series in different ROIs. Hence, the precision matrix **Λ** can contain some non-zero off-diagonal elements. We model this using a Wishart prior for the precision matrix

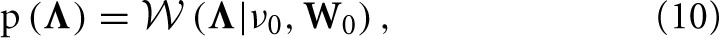



where ν_0_ and **W**_0_ are deterministic parameters. By using a diagonal matrix for **W**_0_, we obtain a prior modeling that encourages **Λ** to be diagonal, which is the structure usually assumed in VAR models. Another reason for chosing this prior modeling is that the Wishart distribution is the conjugate prior for the precision matrix of the Gaussian distribution, which simplifies the inference procedure.

For the observation noise, we assume that the noise in different regions is uncorrelated and use diagonal covariance matrices given by **R** = Diag (**β**)^−1^, where **β** is a precision hyperparameter vector of length *N*. We use conjugate gamma hyperpriors for the precisions as follows

(11) $$\text{p}\left(\beta \right)=\prod_{i=1}^{N}\Gamma \left(\beta_{i}|a_{\beta }^{0},b_{\beta }^{0}\right),$$

where the gamma distribution with shape parameter *a* and inverse scale parameter *b* is given by

(12) $$\Gamma \left(\xi |a,b\right)=\frac{b^{a}}{\Gamma \left(a\right)}\xi^{a-1}\exp\left(-b\xi \right).$$

We usually have some information about the fMRI observation noise and can use this knowledge to set the parameters *a*^0^_β_ and *b*^0^_β_. The setting of the deterministic parameters will be discussed in more detail in the next section.

### 2.4. Global modeling

By combining the probability distribution describing the VAR model, the fMRI observation model, and the prior model, we obtain a joint distribution over all latent variables and known quantities as

(13) $$\begin{array}{l} \text{p}\left(\Theta ,{\left\{y(t)\right\}}_{t=1}^{T}\right)=\left(\prod_{i=1}^{N}\text{p}\left(y_{i}|z_{i},H_{i},\beta_{i}\right)\right)\text{ ​}\left(\prod_{t=1}^{T}\text{p}\left(z(t)|x(t),\vartheta \right)\text{​}\right)\\ \;\times \left(\prod_{t=1}^{T}\text{p}\left(x(t)|x(t-1),{\left\{A^{\left(p\right)}\right\}}_{p=1}^{P},\Lambda \right)\right)\\ \;\times \left(\prod_{p=1}^{P}\text{p}\left(A^{\left(p\right)}|\Gamma \right)\right)\text{p}\left(\Gamma \right)\text{p}\left(\Lambda \right)\text{p}\left(\beta \right), \end{array}$$

where Θ contains all the latent variables of the model, i.e.,

(14) $$\Theta =\left\{{\left\{x(t)\right\}}_{t=1}^{T},{\left\{z(t)\right\}}_{t=1}^{T},{\left\{A^{\left(p\right)}\right\}}_{p=1}^{P},\Gamma ,\;\Lambda ,\;\beta \right\}.$$

**Figure 1:**
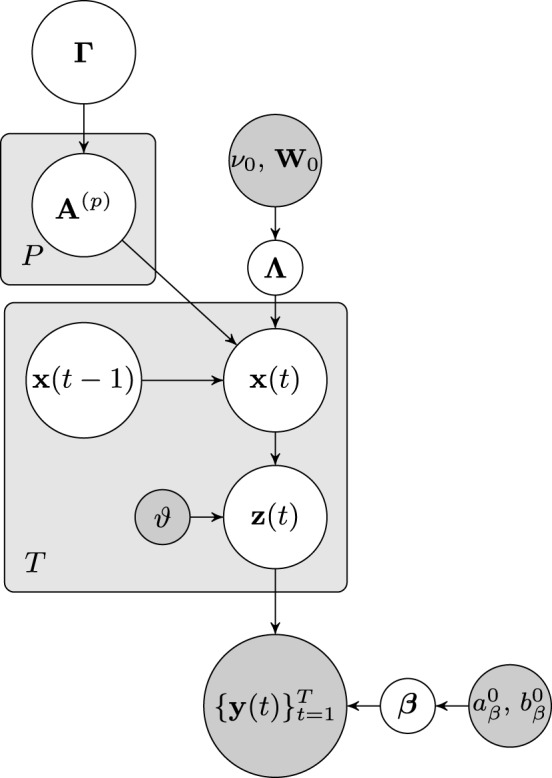
**Graphical model visualizing the dependencies of the joint distribution over the latent variables and the fMRI observations**. Nodes representing latent variables are depicted with white backgrounds while nodes with known quantities have gray backgrounds. Rectangular plates indicate the repetition of nodes.

The dependencies of the joint distribution can be visualized as a directed acyclic graphical model, which is depicted in Figure 1. From the graphical model it can be seen that the node of approximate neuronal time series **z**(*t*) is inserted between the nodes of the neuronal time series **x**(*t*) and the observation **y**(*t*). As will be discussed in the next section, this additional node leads to important computational advantages, as it allows us to separate the hemodynamic deconvolution (estimation of **z**(*t*)) from the estimation of the estimation of the neuronal time series **z**(*t*) and the VAR modeling parameters.

## 3. Bayesian inference

We draw inference based on the posterior distribution

(15) $$\text{p}\left(\Theta |{\left\{y(t)\right\}}_{t=1}^{T}\right)=\frac{\text{p}\left(\Theta ,{\left\{y(t)\right\}}_{t=1}^{T}\right)}{\text{p}\left({\left\{y(t)\right\}}_{t=1}^{T}\right)}.$$

However, as with many probabilistic models, calculating p ({**y**(*t*)}^*T*^_*t* = 1_) and hence calculating the posterior distribution is analytically intractable. Therefore, we approximate the posterior distribution by a simpler distribution using the variational Bayesian (VB) method with the mean field approximation (Jordan et al., 1999; Attias, 2000). For the problem at hand we approximate the posterior by a distribution which factorizes over the latent variables as follows

(16) $$\text{q}\left(\Theta \right)=\text{ q}\left({\left\{x(t)\right\}}_{t=1}^{T}\right)\text{ q}\left({\left\{z(t)\right\}}_{t=1}^{T}\right)\text{ q}\left({\left\{A^{\left(p\right)}\right\}}_{p=1}^{P}\right)\text{ q}\left(\Gamma ,\Lambda ,\beta \right).$$

Using the structure of the graphical model and the property of d-separation, it is found the there are several induced factorizations when assuming the factorization given by Equation (16) (refer to Bishop, 2006 for detailed explanations). We can include the induced factorizations to further factorize to posterior as follows^1^

(17) $$\begin{array}{l} \text{q}\left(\Theta \right)=\text{q}\left({\left\{x(t)\right\}}_{t=1}^{T}\right)\left(\prod_{i=1}^{N}\text{q}\left({\left\{z_{i}(t)\right\}}_{t=1}^{T}\right)\right)\text{q}\left({\left\{A^{\left(p\right)}\right\}}_{p=1}^{P}\right)\\ \;\times \left(\prod_{i=1}^{N}\prod_{k=1}^{N}\text{q}\left(\gamma_{ik}\right)\right)\text{q}\left(\Lambda \right)\left(\prod_{i=1}^{N}\text{q}\left(\beta_{i}\right)\right). \end{array}$$

The key ingredient of this VB method is that we only assume a specific factorization of the posterior but make no assumptions about the functional form of the distributions. Instead, we find the form of each distribution by performing a variational minimization of the Kullback–Leibler (KL) divergence between the approximation and the true posterior. The KL divergence is given by

(18) $$\begin{array}{l} C_{KL}\left(\text{q}\left(\Theta \right)\|\text{p}\left(\Theta |{\left\{y(t)\right\}}_{t=1}^{T}\right)\right)=\\ \;\int \text{q}\left(\Theta \right)\log\left(\frac{\text{q}\left(\Theta \right)}{\text{p}\left(\Theta |{\left\{y(t)\right\}}_{t=1}^{T}\right)}\right)d\Theta \end{array}$$

which is a non-negative measure that is only equal to zero if q (Θ) = p (Θ|{**y**(*t*)}^*T*^_*t* = 1_). A standard result from VB analysis (Bishop, 2006) is that if we express [Equation (17)] as q (Θ) = ∏_*i*_ q(**Φ**_*i*_), i.e., we use q (**Φ**_*i*_) to denote the individual factors in [Equation (17)], the distribution for the *i*-th factor which minimizes [Equation (18)] is given by

(19) $$\ln\text{ q}\left(\Phi_{i}\right)={\langle \ln\text{ p}\left(\Theta ,{\left\{y(t)\right\}}_{t=1}^{T}\right)\rangle }_{\text{q}\left(\Theta \setminus \Phi_{i}\right)}+\text{const},$$

where 〈·〉_q(Θ\**Φ**_*i*_)_ denotes the expectation with respect to distributions q (·) all latent variables except **Φ**_*i*_. Using this, we obtain a distribution for each factor. The VB inference algorithm sequentially updates the sufficient statistics of each distribution until convergence. Below we show the functional form of the variational posterior distribution for each latent variable. Due to space constraints, the derivations are not shown here and we refer to Luessi (2011) for more details.

Using Equation (19), the distribution for the neuronal time series q ({**x** (*t*)}^*T*^_*t* = 1_) is obtained from

(20) $$\begin{array}{l} \ln\text{ q}({\left\{x(t)\right\}}_{t=1}^{T})\\ \;={\langle \ln\overset{T}{\underset{t=1}{\prod }}\text{p}(x(t)|x(t-1),{\left\{A^{\left(p\right)}\right\}}_{p=1}^{P},\Lambda )\;\times \text{p}(z(t)|x(t),\vartheta )\rangle }_{\text{q}({\left\{z(t)\right\}}_{t=1}^{T})\text{q}({\left\{A^{\left(p\right)}\right\}}_{p=1}^{P})\text{q}(\Gamma ,\Lambda ,\beta )}+\text{const}, \end{array}$$

where all terms not depending on {**x** (*t*)}^*T*^_*t* = 1_ have been absorbed into the additive normalization constant. Due to the conjugacy of the priors, q ({**x** (*t*)}^*T*^_*t* = 1_) is a multivariate Gaussian distribution with dimension *TPN*. However, this distribution has a complicated form and cannot be further factorized, which makes a direct calculation of the sufficient statistics computationally infeasible. Note that this complication is not due to the introduction of **z** (*t*); it is also present in methods which do not employ the approximate time series **z**(*t*). Fortunately, Equation (20) has a similar form as an equation encountered in the variational Kalman smoothing algorithm (Beal and Ghahramani, 2001; Ghahramani and Beal, 2001), with the only difference that instead of using the observations we use the expectation of **z**(*t*) under q ({**z** (*t*)}^*T*^_*t* = 1_). The variational Kalman smoothing algorithm recursively estimates q (**x**(*t*)) = 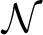
(**x**(*t*)|**μ**_*t*_, **Σ**_*t*_) using a forward and a backward recursion. It is important to point out that we do not introduce an additional factorization of q ({**x** (*t*)}^*T*^_*t* = 1_) over time points, as for example done in Makni et al. (2008), which has been shown to result in an inaccurate approximation to the posterior distribution for large *T* (Wang and Titterington, 2004). Instead, the variational Kalman smoothing algorithm provides an efficient way for estimating q ({**x** (*t*)}^*T*^_*t* = 1_) without assuming a factorization over time points.

In our implementation we ignore the contribution from the covariances in the quadratic terms of {**A**^(*p*)^}^*P*^_*p* = 1_, i.e., we assume 〈(**A**^(*p*)^)^*T*^ (**A**^(*p*)^)〉 = 〈**A**^(*p*)^〉^*T*^ 〈**A**^(*p*)^〉. This assumption is also made in Ryali et al. (2011) and can be expected to have only a minor influence on the performance of the proposed method. The main reason for using this approximation is that we do not need to calculate and store the covariance matrix of q ({**A**^(*p*)^}^*P*^_*p* = 1_), which greatly reduces the computational requirements of the method. Another effect of using this approximation is that the recursive inference algorithm becomes similar to the standard Kalman smoothing algorithm, also known as the Rauch-Tung-Striebel smoother (Rauch et al., 1965). For the forward pass, we use the initial conditions **μ**^0^_0_ = **0**, $\Sigma_{0}^{0}=I$ and calculate for *t* = 1, 2, …, *T* the following

(21) $$\mu_{t}^{t-1}=\langle \tilde{A}\rangle \mu_{t-1}^{t-1}$$

(22) $$\Sigma_{t}^{t-1}=\langle \tilde{A}\rangle \Sigma_{t-1}^{t-1}{\langle \tilde{A}\rangle }^{T}+\langle Q\rangle$$

(23) $$\mu_{t}^{t}=\mu_{t}^{t-1}+K_{t}\left(\langle z\left(t\right)\rangle -B\mu_{t}^{t-1}\right)$$

(24) $$\Sigma_{t}^{t}=\Sigma_{t}^{t-1}-K_{t}B\Sigma_{t}^{t-1},$$

where the Kalman gain is given by

(25) $$K_{t}=\Sigma_{t}^{t-1}B^{T}{\left(B\Sigma_{t}^{t-1}B^{T}+\vartheta^{-1}I_{N}\right)}^{-1}.$$

After the forward pass, the final estimate for the last time point has been obtained, i.e., we have **μ**_*T*_ = **μ**^*T*^_*T*_ and $\Sigma_{T}=\Sigma_{T}^{T}$. For the remaining time points we execute a backward pass and calculate the sufficient statistics of q (**x**(*t*)) for *t* = *t* − 1, *t* − 2, …, 1 as follows

(26) $$\mu_{t}=\mu_{t}^{t}+J_{t}\left(\mu_{t+1}-\langle \tilde{A}\rangle \mu_{t}^{t}\right),$$

(27) $$\Sigma_{t}=\Sigma_{t}^{t}+J_{t}\left(\Sigma_{t}^{t}-\Sigma_{t+1}^{t}\right)J_{t}^{T},$$

where

(28) $$J_{t}=\Sigma_{t}^{t}{\langle \tilde{A}\rangle }^{T}{\left(\Sigma_{t+1}^{t}\right)}^{-1}.$$

As the posterior distributions of individual time points are not independent, i.e., q ({**x** (*t*)}^*T*^_*t* = 1_) ≠ ∏^*T*^_*t* = 1_ q (**x**(*t*)), cross-time expectations contain a cross-time covariance **Σ**_*t,t* − 1_, i.e., 〈**x**(*t*)**x**(*t* − 1)^*T*^〉 = **μ**_*t*_ **μ**^*T*^_*t* − 1_ + **Σ**_*t,t* − 1_. Such cross-time covariance terms are computed as follows (see Ghahramani and Hinton, 1996)

(29) $$\Sigma_{t,t-1}=\Sigma_{t}J_{t-1}^{T}+J_{t}\left(\Sigma_{t+1,t}-\langle \tilde{A}\rangle \Sigma_{t}^{t}\right)J_{t-1}^{T}.$$

The posterior distribution of the approximate time series for the *i*-th region q ({*z*_*i*_(*t*)}^*T*^_*t* = 1_) is found to be a Gaussian, that is,

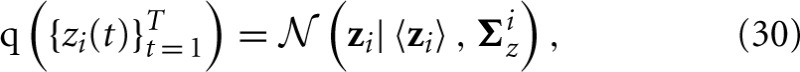



with parameters

(31) $$\langle z_{i}\rangle =\Sigma_{z}^{i}\left(\langle \beta_{i}\rangle H_{i}^{T}y_{i}+\vartheta \langle x_{i}\rangle \right),$$

(32) $$\Sigma_{i}^{z}={\left(\langle \beta_{i}\rangle H_{i}^{T}H_{i}+\vartheta I_{T}\right)}^{-1}.$$

The distribution for the VAR coefficients **a** = vec ([**A**^(1)^ **A**^(2)^ ··· **A**^(*P*)^]) is also Gaussian, the mean and covariance matrix are given by

(33) $$\langle a\rangle =\Sigma_{a}\text{vec}\left(\langle \Lambda \rangle \left[\sum_{t=1}^{T}{\left(\mu_{t}\right)}_{1:N}\mu_{t-1}^{T}+{\left(\Sigma_{t,t-1}\right)}_{1:N,:}\right]\right)\;$$

(34) $$\Sigma_{a}^{-1}=P_{1}⊗\langle \Lambda \rangle +\text{ Diag}\left(I_{P}⊗\text{vec}\left(\langle \Gamma \rangle \right)\right),$$

where the matrix **P**_1_ is given by

(35) $$P_{1}=\sum_{t=1}^{T}\langle x(t-1)x{\left(t-1\right)}^{T}\rangle =\sum_{t=1}^{T}\mu_{t-1}\mu_{t-1}^{T}+\Sigma_{t-1}.$$

Notice that the size of $\Sigma_{a}^{-1}$ is *N*^2^*P* × *N*^2^*P*. Hence, for large *N* performing a direct inversion is computationally very demanding and potentially numerically inaccurate. Moreover, storing the matrix requires large amounts of memory. Instead of directly inverting the matrix, we use a conjugate gradient (CG) algorithm to solve

(36) $$\Sigma_{a}^{-1}\langle a\rangle =\text{vec}\left(\langle \Lambda \rangle \left[\sum_{t=1}^{T}{\left(\mu_{t}\right)}_{1:N}\mu_{t-1}^{T}+{\left(\Sigma_{t,t-1}\right)}_{1:N,:}\right]\right)\text{​},\;$$

for 〈**a**〉, which is possible since $\Sigma_{a}^{-1}$ is symmetric positive definite. The CG algorithm only needs to compute matrix-vector products of the form $\Sigma_{a}^{-1}p$. From the structure of $\Sigma_{a}^{-1}$, one can see that the multiplication of the diagonal matrix on the right side is simply the element-wise product of the diagonal of Diag (**I**_*P*_ ⊗ vec (〈**Γ**〉)) and **p**, which can be computed efficiently. Similarly, (**P**_1_ ⊗ 〈**Λ**〉) **p** can be computed efficiently without computing the Kronecker product (Fernandes et al., 1998).

Note that computation of the gamma hyperparameters requires access to the diagonal elements of **Σ**_*a*_. Since we do not explicitly compute **Σ**_*a*_, we approximate the diagonal by $\text{Diag}\left(\Sigma_{a}\right)\approx \text{Diag}{\left(\text{diag}\left(\Sigma_{a}^{-1}\right)\right)}^{-1}$. We performed experiments with small *N* where we calculated **Σ**_*a*_ directly using a matrix inversion. We found that using the CG algorithm with an approximation to the diagonal of the covariance matrix results in virtually the same estimation performance for the proposed method, while being much faster and more memory efficient.

The posterior for the noise precision **Λ** is Wishart distributed with q (**Λ**) = 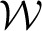 (**Λ**|ν, **W**) where the parameters are given by

(37) $$\nu =T+\nu_{0},$$

(38) $$W^{-1}=\langle P_{2}\rangle +W_{0}^{-1}.$$

The expectation 〈**P**_2_〉 is given by

(39) $$\begin{array}{l} \langle P_{2}\rangle =\sum_{t=1}^{T}\left({\left(\mu_{t}\right)}_{1:N}-\langle \bar{A}\rangle \mu_{t-1}\right){\left({\left(\mu_{t}\right)}_{1:N}-\langle \bar{A}\rangle \mu_{t-1}\right)}^{T}\\ \;-{\left(\Sigma_{t,t-1}\right)}_{1:N,:}\langle \bar{A}\rangle -{\langle \bar{A}\rangle }^{T}{\left(\Sigma_{t,t-1}\right)}_{1:N,:}^{T}\\ \;+{\left(\Sigma_{t}\right)}_{1:N,1:N}+\langle \bar{A}\rangle \Sigma_{t-1}{\langle \bar{A}\rangle }^{T}, \end{array}$$

where **A** = [**A**^(1)^ **A**^(2)^ ··· **A**^(*P*)^], (**Σ**_*t*_)_1:*N*,1:*N*_ is the top left *N* × *N* block of **Σ**_*t*_, and (**Σ**_*t,t* − 1_)_1:*N*,:_ are the first *N* rows of **Σ**_*t,t* − 1_. The mean of the Wishart distribution is given by 〈**Λ**〉 = ν**W**, which is the value used in the other distribution updates in the VB algorithm.

The distribution for the VAR precision hyperparameter q (γ_*ij*_) is found to be a gamma distribution with shape and inverse scale parameters

(40) $$a_{\gamma }^{i,j}=\frac{P}{2},\;b_{\gamma }^{i,j}=\frac{1}{2}\sum_{p=1}^{P}\left({\langle a_{ij}^{\left(p\right)}\rangle }^{2}+{\bar{a}}_{ij}^{\left(p\right)}\right),$$

where *ā*^(*p*)^_*ij*_ is the variance of *a*^(*p*)^_*ij*_, which we obtain from the approximation to the diagonal of **Σ**_*a*_. Similarly, the posterior for the observation noise precision is a gamma distribution with the following shape parameter aβi= T2+aβ0 and inverse scale parameter

(41) $$b_{\beta }^{i}=\frac{1}{2}[y_{i}^{T}y_{i}-2y_{i}^{T}H_{i}\langle z_{i}\rangle +{\langle z_{i}\rangle }^{T}H_{i}^{T}H_{i}\langle z_{i}\rangle +\text{tr}\left(H_{i}^{T}H_{i}\Sigma_{i}^{z}\right)]+b_{\beta }^{0}.$$

### 3.1 Selection of deterministic parameters

The proposed method has several deterministic parameters which have to be specified by the user, namely, the observation noise precision parameters {*a*^0^_β_, *b*^0^_β_}, the VAR model noise parameters {ν_0_, **W**_0_}, and the neuronal approximation precision ϑ. Typically, an estimate of the noise variance σ^2^ present in the data is available to the user. If this case, a reasonable setting of the observation noise precision parameters is *a*^0^_β_ = *c*, *b*^0^_β_ = *c*σ^2^, where *c* is a constant related to the confidence in our initial noise estimate. For very small values of *c*, the observation noise precision will be estimated solely by the algorithm, while a high value forces the estimated noise precision to the value specified by the user. Unless otherwise noted, we assume throughout this work that an estimate of the noise variance is available and use *c* = 10^9^.

On the other hand, the user typically does not have precise *a priori* knowledge of the AR innovation precision. In this case, one option is to use ν_0_ = 0, **W**^−1^_0_ = **0**, which is equivalent to an non-informative Jeffreys prior for the AR innovation precision matrix. However, we observed that 〈**Λ**〉 can attain values that are too large when a non-informative prior is used. This behavior is caused by the fact that the convolution with the HRF acts as a low-pass filter and it is generally not possible to perfectly recover the high frequency content of the neuronal signal, causing an over-estimation of the AR innovation precision. We found that using ν_0_ = 1 and **W**_0_ = 10^−3^**I**, prevents 〈**Λ**〉 from attaining too large values and we use this setting in all experiments presented in this work. Naturally, the parameter setting depends on the scale of the fMRI observation. Throughout this work, we rescale the fMRI observation to have an RMS value of 6.0, where the root-mean-square (RMS) value is calculated as $\text{RMS}=\sqrt{\left(\sum_{t=1}^{T}\|y\left(t\right){\|}_{2}^{2}\right)/\left(NT\right)}$. Note that the choice of RMS = 6.0 is arbitrary, i.e., different values could be used but then other deterministic parameters would have to be modified accordingly. Finally, the approximation precision parameter ϑ plays an important role. In Equation (32) it acts similarly to a regularization parameter while having the role of the observation noise precision in the variational Kalman smoother. We heuristically found that using a value that is higher than the observation noise precision works well and we use ϑ = 10/σ^2^ throughout this work.

### 3.2. Computational advantages of the proposed approach

To conclude this section, we highlight some important advantages in terms of computational requirements of the proposed method over previous approaches. The advantages of the proposed method are directly related to the introduction of the approximate time series **z**(*t*).

The first advantage is due to the separation of the model of the neuronal time series from the hemodynamic convolution model, which leads to a reduced state-space dimension of the Kalman smoothing algorithm. More specifically, in Smith et al. (2010); Ryali et al. (2011), the observation process is modeled as

(42) $$y(t)=\tilde{H}x(t)+\varepsilon \left(t\right),$$

where 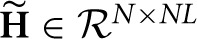 is a matrix that contains the HRFs of all regions. This modeling requires that **x**(*t*) is an embedding process over *L* time points, i.e., the dimension of **x**(*t*) is *D* = *NL*, as opposed to *D* = *NP* in our method. The higher dimension leads to excessive memory requirements as the state-space dimension of the Kalman smoothing algorithm is increased and a total of 2*T* covariance and cross-time covariance matrices of size *D* × *D* need to be stored in memory. As an example, assuming double precision floating point arithmetic and *P* = 2, *L* = 20, *N* = 100, *T* = 1000, the methods in Smith et al. (2010) and Ryali et al. (2011) require approximately 60 GB of memory to store the covariance matrices, whereas the proposed method only requires approximately 600 MB. The large memory consumption and the higher dimension of the required matrix inversions is the reason why previous methods become computationally infeasible for large scale problems where *N* ≈ 100 and *T* ≈ 1000. The problem is even more severe for low TR values, since the HRF typically has a length of about 30 s and a higher sampling rate means more samples are needed to represent the HRF, thus increasing the value of *L*.

The second advantage due to introduction of **z**(*t*) is that the approximate posterior of **z**(*t*) factorizes over ROIs and we can update the posterior distribution q ({*z*_*i*_(*t*)}^*T*^_*t* = 1_) for each region separately using Equations (31, 32). For large numbers of time points this computation can still be expensive as the inversion of a *T* × *T* matrix is required. However, notice that if we assume that the convolution with **h**_*i*_ is circular, the matrix **H**_*i*_ becomes circulant. Circulant matrices can be diagonalized by the discrete Fourier transform (see, e.g., Moon and Stirling, 2000). Hence, it is possible to perform the calculation of 〈**z**_*i*_〉 in the frequency domain. In our implementation we use a fast Fourier transform (FFT) algorithm with zero-padding such that the circular convolution corresponds to a linear convolution. The resulting time complexity is *O*(*T* log *T*), compared to *O*(*T*^3^) when a direct matrix inversion is used. Moreover, notice that $\Sigma_{i}^{z}$ is circulant as well, which allows us to reduce the computational and memory requirements by only calculating and storing the first row of $\Sigma_{i}^{z}$ (all other rows can be obtained by circular shifts of the first row).

## 4. Empirical evaluation with simulated data

In this section, we evaluate the performance of the proposed method using a number of different simulation scenarios. In all simulations, the proposed method is denoted by “VBCCA” (Variational Bayesian Causal Connectivity Analysis). For comparison purposes we include the conditional WGC analysis method implemented in the “Granger Causal Connectivity Analysis (GCCA) toolbox” (Seth, 2010), which we denote by “WGCA” (Wiener–Granger Causality Analysis). Note that we use WGCA for comparison as it is a widely used method with publicly available implementations. More recent methods, such as the methods from, Smith et al. (2010), Marinazzo et al. (2011) and Ryali et al. (2011) may offer a higher estimation performance than WGCA. However, their high computational complexity makes it difficult to apply them to large-scale problems, which is the situation where our method clearly outperforms WGCA. Nevertheless, we include a comparison with a modified version of our method, which does not use an approximation to the neuronal time series and is therefore more similar to the method from Ryali et al. (2011), and show that for small networks our method provides a comparable estimation performance.

### 4.1. Quality metrics

We use two objective metrics to evaluate the performance of the methods. The first metric serves to quantify the performance in terms of correctly detecting the presence of a connection between regions, without taking the direction of the causal influence into account. In order to do so, we calculate the area under the receiver operating characteristic (ROC) curve, which is commonly used in signal detection theory and has also previously been used to evaluate connectivity methods (Valdés-Sosa et al., 2005; Haufe et al., 2008). In the following we give a short explanation of the ROC curve and refer the reader to Fawcett (2006) for a more detailed introduction. The ROC curve is generated by applying thresholds to the estimated connectivity scores. The resulting binary masks are compared with the ground truth, resulting in a number of true positives (TP) and false positives (FP). From the TP and FP numbers, we can calculate the true positive rate (TPR) and false positive rate (FPR) as follows

(43) $$\text{TPR}=\frac{\text{TP}}{\text{P}},\text{ FPR}=\frac{\text{FP}}{\text{N}},$$

where *P* and *N* are the total number of positives and negatives, respectively. For each threshold, we obtain a (FPR, TPR) point in the ROC space. By applying all possible thresholds, we can construct the ROC curve which allows us to compute the area under the curve (AUC). The AUC is the metric used here to evaluate the connection detection performance. The value of the AUC is on the interval [0 1], with 1.0 being perfect detection performance while 0.5 is the performance of a random detector, i.e., the AUC should always be above 0.5 and as close as possible to 1.0. To calculate the non-directional connectivity score between nodes *i* and *j* from the estimated *N* × *N* connectivity matrix, we use the larger of the directional scores, i.e., con(*i, j*) = con(*j, i*) = max (*c*_*ij*_, *c*_*ji*_). For WGCA, the matrix **C** is the matrix with estimated Granger causality scores, whereas for the proposed method we calculate **C** from the estimated VAR coefficients using $c_{ij}=\sqrt{\sum_{p=1}^{P}\langle a_{ij}^{\left(p\right)}\rangle }$.

The AUC provides information on the performance in terms of detecting connections without taking directionality into account. A second metric, denoted by “d-Accuracy” (Smith et al., 2011), is used to evaluate the ability of a method to correctly identify the direction of the connection. The d-Accuracy is calculated as follows. For true connections (known from the ground truth) we compare the elements *c*_*ij*_ and *c*_*ji*_ in the connectivity matrix. We decide that the direction was estimated correctly if *c*_*ij*_ > *c*_*ji*_ and the true connection has the direction *j* → *i*. By repeating for all connections, we calculate the overall probability that the direction was estimated correctly, which is the d-Accuracy score. Like the AUC, the d-Accuracy lies between 0 and 1 with 1.0 indicating perfect performance and 0.5 being the performance of a random directionality detector.

### 4.2. Network size and SNR

In this experiment we evaluate the performance of the proposed method for a number of networks of varying sizes and a number of different signal-to-noise ratios (SNRs). We generate neuronal time series according to Equation (1) where we simulate connectivity by randomly activating ⌈*N*/2⌉ uni-directional connections, for which we generate the VAR coefficients according to *a*^(*p*)^_*ij*_ ~ 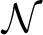
(0, 0.05) ∀ *p* ∈ {1, …, *P*}, with *P* = 2. The noise term is chosen to be Gaussian with unit variance, i.e., **η**(*t*) ~ 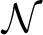
(0, **I**_*N*_). Using the VAR coefficient matrices we generate a neuronal time series **s** (*t*) with a total of *T* = 500 time points. To generate the fMRI observations, we convolve the neuronal time series of each node with the canonical HRF implemented in SPM8 (http://www.fil.ion.ucl.ac.uk/spm/), which has a positive peak at 5 s and a smaller negative peak at 15.75 s. The HRF used has a total length of 30 s assuming a sampling rate of 1 Hz (*L* = 30). Finally, to generate the noisy fMRI observation **y** (*t*), we add zero-mean, independent, identically distributed (i.i.d.) Gaussian noise with a variance σ^2^ determined by the SNR used, i.e., ${\text{SNR}}_{\text{dB}}=10\log_{10}\left(\left(\sum_{t=1}^{T}\|y\left(t\right)-\bar{y}\left(t\right){\|}_{2}^{2}\right)/(NT\sigma^{2})\right)$, where **y** (*t*) is the observation without additive noise.

**Figure 2:**
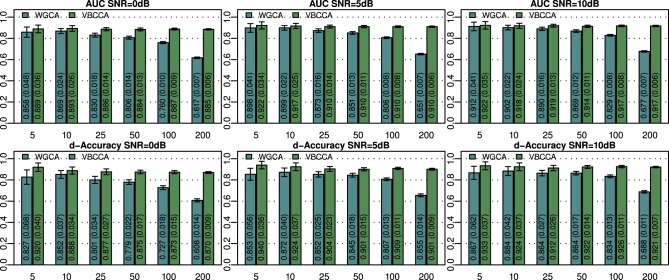
**Area under ROC curve (AUC) and d-Accuracy scores for random networks with sizes between 5 and 200 nodes and different SNRs**. The proposed method is denoted by VBCCA, whereas WGCA denotes Wiener–Granger causality analysis. All results are averages over 50 simulations with error bars indicating the 95% confidence intervals. The average scores are also shown as numerical values in the bar plot, where the values in parentheses are the size of one side of the confidence interval.

The simulated noisy observations are used as inputs to the evaluated connectivity methods. In this experiment we use the true VAR order, i.e., *P* = 2, for each evaluated method. Additionally, in the proposed method we use the same canonical HRF that is used to generate the data. Results for networks with *N* = {5, 10, 25, 50, 100, 200} nodes and SNRs of 0, 5, and 10 dB are shown in Figure 2. For small networks (5 and 10 nodes) both methods offer similar performance with the proposed method being slightly better. The SNR has a small influence on the performance and it can be concluded that each method performs similarly across the SNRs shown. As expected, the performance of both methods decreases with increasing network size. However, WGCA is affected drastically compared to the proposed method, which shows almost constant performance across network sizes. The proposed method clearly outperforms WGCA for large networks (more than 25 nodes). For *N* = 200, the AUC for WGCA is approximately 0.65, which is very poor. Therefore, for the given number of time samples, it can be concluded that WGCA is not suitable for connectivity analysis in large scale networks.

### 4.3. VAR order

**Figure 3:**
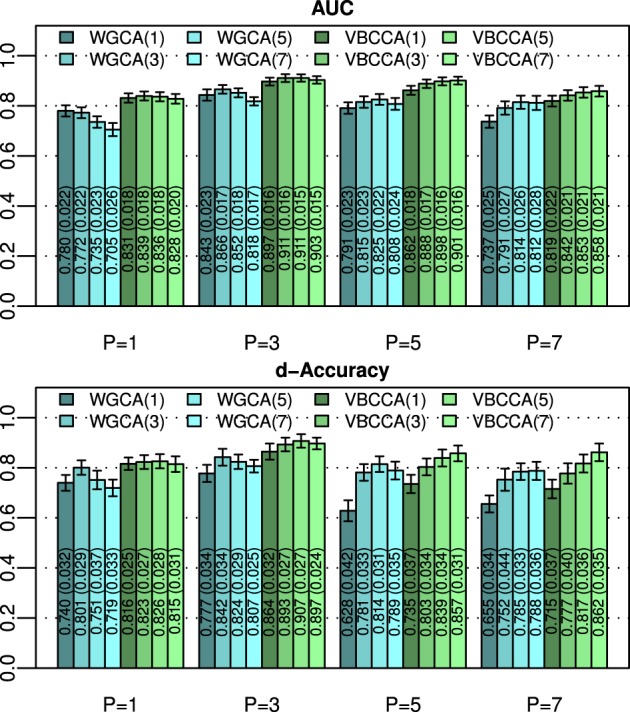
**Area under ROC curve (AUC) and d-Accuracy scores for data generated by VAR processes with orders from 1 to 7**. The proposed method and the Wiener–Granger causality method are denoted by VBCCA(*P*) and WGCA(*P*), respectively, where *P* denotes the VAR model order used in the algorithm. All results are averages over 50 simulations with error bars indicating the 95% confidence intervals. The average scores are also shown as numerical values in the bar plot, where the values in parentheses are the size of one side of the confidence interval.

An important question is how the performance is affected by a mismatch in the VAR order present in the data and the VAR order assumed in the algorithm. For this evaluation we generate simulated data using the same procedure as in the first experiment for *N* = 25 and an SNR of 0 dB, but we vary the VAR order from 1 to 7. The generated data is used as input to the evaluated methods for which we vary the VAR order used in the algorithm in the same range, i.e., from 1 to 7. Results for this simulation are shown in Figure 3; it can be seen that the proposed method typically outperforms the WGCA method even if there is a mismatch between the VAR order in the data and the VAR order used in the algorithm. It is also interesting to note that the proposed method typically performs well as long as the VAR order used in the algorithm is equal or higher than that present in the data. This behavior can be attributed to two factors. First, the proposed method employs a grouping of VAR coefficients across lags through shared priors, which limits the model complexity even when the VAR order is increased. Second, we use an approximation to the posterior distribution to estimate the VAR coefficients; it is well known that methods which draw inference based on the posterior distribution are less prone to over-fitting than other methods, such as, maximum likelihood methods.

### 4.4. Effect of using an approximation to the neuronal signal

**Figure 4:**
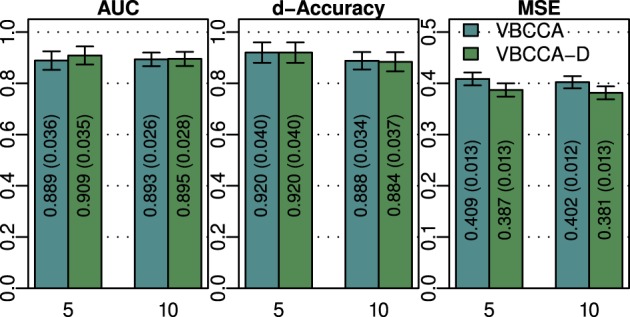
**AUC, d-Accuracy, and mean squared error (MSE) scores for the proposed method with and without using the approximate time series **z** (*t*)**. The method are denoted by VBCCA (**z** (*t*) used) and VBCCA-D (**z** (*t*) not used). The simulation parameters are the same as in the first experiment, i.e., *N* = {5,10}, *T* = 500, *P* = 2, SNR = 0 dB. All results are averages over 50 simulations with error bars indicating the 95% confidence intervals. The average scores are also shown as numerical values in the bar plot, where the values in parentheses are the size of one side of the confidence interval.

**Figure 5:**
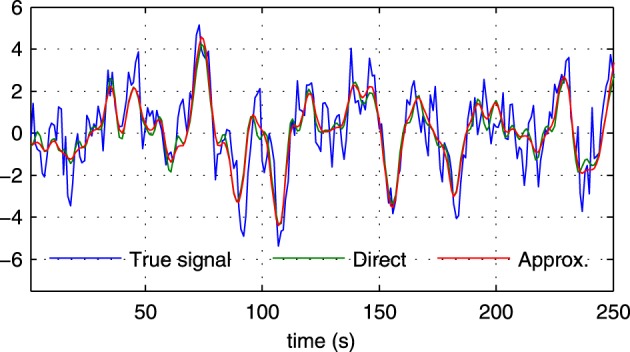
**Sections of the true neuronal signal (blue) and estimated neuronal signals for one node in a simulation with *N* = 5, SNR = 0 dB in the first experiment**. The neuronal signal estimated by the proposed method is shown in red (“Approx.”), while the neuronal signal estimated by the proposed method without using the approximate time series **z** (*t*) is shown in green (“Direct”).

As discussed in previous sections, the proposed method employs a hierarchical Bayesian model with an approximation to the neuronal time series. The approximate time series is denoted by **z**(*t*) and is a key part of the proposed method as it enables the method to be computationally efficient through a reduction of the state space dimension used in the Kalman smoother. In addition, the time series **z**(*t*) can be efficiently estimated in the frequency domain using fast Fourier transform algorithms. While the introduction of this approximation improves the computational efficiency, some reduction in the estimation performance may be caused. To quantify the influence of this approximation, we have implemented a modified version of the proposed method where **z**(*t*) is not used, i.e., we increase the dimension of **x**(*t*) to *D* = *NL* and model the observation process using Equation (42). This part of the modified model exactly corresponds to what is used in Smith et al. (2010) and Ryali et al. (2011). Due to the excessive memory requirements, the modified version of the proposed method, which we denote by “VBCCA-D,” can only be used for networks with small numbers of regions and HRFs consisting of a small number of time samples. We apply the method to the same data that is used in the first experiment, with *N* = {5, 10}, SNR = 0 dB. The resulting connectivity scores, as well as, the mean squared error (MSE) of the neuronal signal are shown in Figure 4. The MSE is calculated as follows

(44) $$\text{MSE}=\left[\sum_{t=1}^{T}\|s\left(t\right)-\tilde{s}\left(t\right){\|}_{2}^{2}\right]/\left[\sum_{t=1}^{T}\|s\left(t\right){\|}_{2}^{2}\right],$$

where **s** (*t*) and $\tilde{s}\left(t\right)$ are the true and the estimated neuronal signals, respectively. It can be seen that the use of the neuronal approximation does not have a negative influence on the performance in terms of AUC while the MSE is slightly lower when the approximation is not used. The small difference in terms of MSE implies that both methods estimate the neuronal signal with similar estimation quality. This is also apparent from Figure 5, which shows the time neuronal series for one region estimated with and without the approximation.

### 4.5. Downsampling and HRF variations

**Figure 6:**
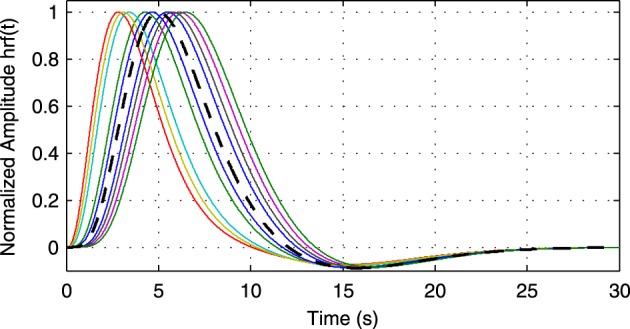
**Example of random hemodynamic response functions (HRFs) used in the experiment**. The HRFs are generated from canonical HRFs where the parameters are drawn from a uniform distribution such that positions of the positive and the negative peaks lie in the intervals [2.5s, 6.5s] and [15s, 16.7s], respectively. The bold dashed line shows the default HRF with peaks at 5 and 15.75 s.

As processing at the neuronal level occurs at temporal scales which are orders of magnitudes faster than the sampling interval of the MRI scanner, it is important to analyze how the performance of causality based methods is affected by the low sampling rate. Another important question is the effect of HRF variability on the performance. In this experiment we analyze the influence of these effects on the estimated causality. In order to do so, we generate **s**(*t*) for two regions and a single connection according to Equation (1) with zero-mean, i.i.d., Gaussian innovations, i.e., **η** (*t*) ~ 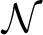 (0, **I**). The simulated sampling rate at the neuronal level is 1 kHz and we generate a total of 240 s of data. We use *a*^1^_1,1_ = *a*^1^_2,2_ = 0.95 to simulate a degree of autocorrelation within each time series. To simulate connection with a certain neuronal delay, depending of the direction of the influence we draw the value of either *a*^*d*^_1,2_ or *a*^*d*^_2,1_ from a uniform distribution on the interval [0.4, 0.9]. The lag parameter *d* is used to simulate the neuronal delay, e.g., *d* = 10 corresponds to a delay of 10 ms. Next, we convolve the obtained neuronal time series with an HRF for each region. In the first simulation we use the same canonical HRF with peaks at 5 and 15.75 s for both regions, whereas in the second simulation we use a randomly generated HRF for each region. To generate a random HRF, we use the HRF generation function provided in SPM8 (http://www.fil.ion.ucl.ac.uk/spm/). The parameter controlling the time-to-peak is drawn from a uniform distribution, such that the positions of the positive peak lies between 2.5 and 6.5 s, which is the range of peak positions reported in Handwerker et al. (2004). The parameter controlling the position of the negative peak (“undershoot”) is held constant at 16 s. Due the implementation in SPM8, the negative peak of the generated HRF lies between 15 and 16.7 s, depending on the position of the positive peak. An example of HRFs used in our experiment is depicted in Figure 6. After each time series has been convolved with a HRF, the data is downsampled to simulate a certain TR value. Finally we add zero-mean, i.i.d., Gaussian noise such that the resulting SNR is 0 dB. To study both the influence of downsampling and the neuronal delay, we linearly vary the simulated TR between 50 ms and 2 s using a step size of 50 ms (40 points) and the delay using 40 linearly spaced values between 5 and 300 ms, resulting in a total of 1600 TR/delay combinations.

**Figure 7:**
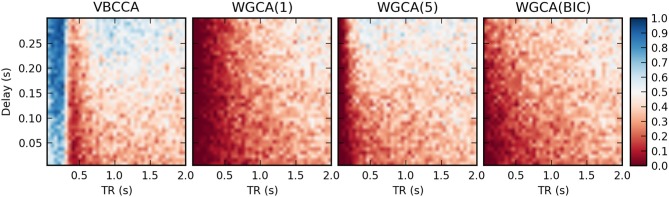
**Average d-Accuracy calculated over 50 simulations for a network with two nodes and a single connection for varying neuronal delays (40 steps between 5 and 300 ms) and TR values of the fMRI scanner (40 steps between 50 ms and 2 s)**. The HRF is held constant for all simulations, the signal-to-noise ratio is 0 dB. The proposed method is denoted VBCCA and we use *P* = 1, whereas WGCA(*P*) denotes the Wiener–Granger causality method, for which we use AR model orders of 1, 5, and an order between 1 and 20 selected using the Bayesian information criterion (BIC).

Results for the first simulation, in which the HRF is held constant, are shown in Figure 7. The results confirm previous findings (Seth et al., 2013) that downsampling confounds WGC. One might intuitively expect that when the neuronal delay is held constant, a lower TR will lead to a higher d-Accuracy. However, our simulations show that this is not necessarily the case; For very low delay and TR values, the WGCA method has d-Accuracy to zero, i.e., it consistently estimates a causal influence with the opposite direction of the true influence, while it approaches the chance level (0.5) when TR is increased. The proposed method shows a similar behavior, but for TR values below 300 ms the d-Accuracy is close to 1.0. While it is difficult to assess the origin of this transition, it is likely caused by increased aliasing that occurs for larger TR values. Together with the consistent causality inversion of WGC for low TR values, it shows that causal information is still present in the data.

**Figure 8:**
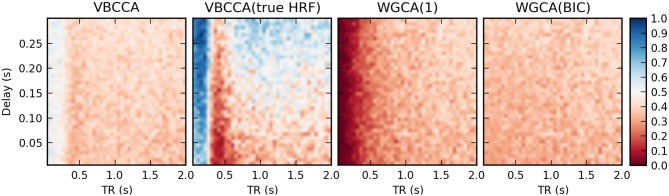
**Average d-Accuracy calculated over 50 simulations for a network with two nodes and a single connection for varying neuronal delays (40 steps between 5 and 300 ms) and TR values of the fMRI scanner (40 steps between 50 ms and 2 s)**. Random HRFs are used with a time-to-peak uniformly distributed between 2.5 and 6.5 s, as shown in Figure 6, the signal-to-noise ratio is 0 dB. The proposed method is denoted VBCCA and we use *P* = 1, VBCCA(true HRF) denotes the proposed method with *P* = 1 and the HRF assumed in the algorithm is the same as the HRF that was used to generate the data. WGCA(*P*) denotes the Wiener–Granger causality method, for which we use AR model orders of 1 and an order between 1 and 20 selected using the Bayesian information criterion (BIC).

In the second simulation, we additionally introduce HRF variations. Results are shown in Figure 8. In this case, the proposed method performs poorly, even for low TR values, unless the method is provided with the true HRF for each region, in which case it can mitigate the effects of HRF variability. Somewhat surprisingly, WGCA(1) performs similarly as before when the same HRF was used for each region. However, when the BIC is used to determine the model order, the WGCA method exhibits low estimation performance for all TR and delay values. A possible explanation for this behavior is that due to the HRF convolution, the selected model order is higher than the true order and the order also depends on the HRF used (Seth et al., 2013), which results in spurious causality inversions and hence poor performance.

**Figure 9:**
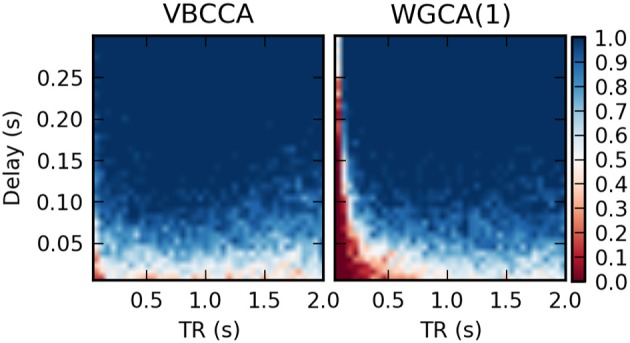
**Average d-Accuracy calculated over 50 simulations for a network with two nodes and a single connection for varying neuronal delays (40 steps between 5 and 300 ms) and TR values of the fMRI scanner (40 steps between 50 ms and 2 s)**. The HRF is held constant for all simulations, the signal-to-noise ratio is 20 dB. The proposed method is denoted VBCCA and we use *P* = 1, whereas WGCA(1) denotes the Wiener–Granger causality method, for which we also use AR model order of 1.

It is important to point out that our results should not be interpreted in the way that WGC with a fixed model order consistently estimates a causal influence with the opposite direction for low TR values; whether the inversion occurs is dependent on simulation parameters, e.g., the amount of autocorrelation in the simulated time series, the connection strength, and the signal-to-noise ratio. For example, when we repeat the first simulation with a higher signal-to-noise ratio of 20 dB, the results change drastically, as shown in Figure 9. The WGCA method now correctly estimates the direction of the influence except for low TR and delay values. In this case also the proposed method performs poorly for low delay values. These results show that while the proposed method performs better, especially in low-SNR situations, there is a risk of causality inversion for both methods. The superiority of the proposed method can be explained by the modeling, which explicitly takes additive noise into account. However, at the same time, both the proposed method and the WGCA method do not model the non-linear downsampling operation and therefore can fail to correctly estimate the direction of the causal influence when the data has been downsampled.

## 5. Application to fMRI data

In this section, we apply the proposed method to resting-state fMRI data provided by the Human Connectome Project (HCP) (Van Essen et al., 2012). We use data from two 15 min runs of the same subject (100307), each consisting of 1200 volumes with a TR of 0.7 s. The minimally preprocessed volume data (Glasser et al., 2013) was aligned to the FreeSurfer (Fischl, 2012) “fsaverage” template and data from 148 cortical parcels from the Destrieux atlas (Destrieux et al., 2010) was extracted by averaging data across the gray matter at each vertex of the FreeSurfer surface mesh. In addition, we extracted volume data from six subcortical parcels (thalamus, caudate, putamen, pallidum, hippocampus, amygdala) for each hemisphere, resulting in a total of 160 parcels. The extracted data was further preprocessed to reduce motion artifacts, slow drifts, and physiological artifacts. Specifically, we reduced motion artifacts and slow drifts using a linear regression for each voxel time series with three motion parameters and a cosine basis up to order 8 as nuisance regressors, where the order of the cosine basis was determined using the Bayesian Information Criterion (BIC) (Schwarz et al., 1978). To reduce physiological noise, we used a procedure similar to CompCor (Behzadi et al., 2007), i.e., we extracted data from the left and right lateral ventricles, which can be expected to not contain any signal of neuronal origin, applied the previously described detrending and motion artifact correction to it, and finally used a principal component analysis (PCA) to extract the 20 strongest temporal components. The extracted noise components were then used as nuisance regressors for each voxel time series where the number of components to use was determined using BIC. Finally, to obtain a single time series for each parcel, we computed a PCA for the data within each parcel and retained the first principal component.

**Figure 10:**
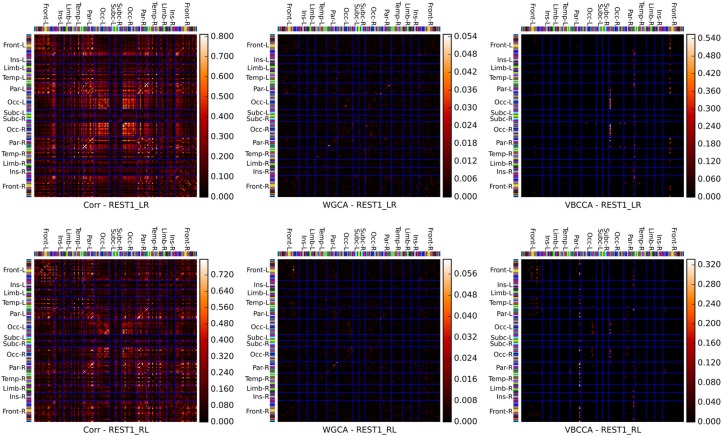
**Connectivity matrices showing the absolute correlation coefficient (Corr), Wiener–Granger causality (WGCA), and causality estimated by the proposed method (VBCCA)**. We use the same parcel grouping and order as in Irimia et al. (2012), which groups the parcels into cortical lobes, i.e., frontal (Front), insular (Ins), limbic (Lim), temporal (Temp), parietal (Par), occipital (Occ), and subcortical (Subc). The “−L” and “−R” suffixes indicate the left and right hemisphere, respectively. The parcel colors are the same as in the standard FreeSurfer color table. Results for the first run (REST1_LR) and the second run (REST1_RL) are in the top and bottom row, respectively. For WGCA and VBCCA, we use an VAR order of *P* = 1 consistent with our simulations. For the proposed method we show $\sqrt{c_{ij}}$ in order to better depict the estimated values within the scale of the color map.

Connectivity matrices obtained by applying the proposed method and WGCA to the HCP data are shown in Figure 10. As a reference we also include the correlation coefficient, which is the most commonly used fMRI resting-state connectivity measure. All methods show some consistency across runs. For the proposed method and the second run, it can clearly be seen that the method finds connections between nodes that are commonly associated with resting-state networks. For example, nodes in the frontal cortices, the temporal lobes, and the parietal lobes, which are part of the default-mode network (Raichle et al., 2001). There is also strong bi-lateral connectivity between the left- and right occipital cortices, which are part of the visual resting-state network. Compared to correlation and WGCA, the VBCCA connectivity matrices are very sparse, which could indicate that there may not be enough causal information in the data to result in strong causality estimates, which would be a sensible explanation given the short propagation delays at the neuronal level and the still relatively slow sampling interval of 0.7 s. Finally, it is important to note that due to the methodological problems discussed in the previous section, it is possible that the direction of the causal influence is estimated incorrectly. The application to real fMRI data as presented here serves as a demonstration, further evaluations, e.g., using simultaneous EEG and fMRI data, are necessary to quantify the effectiveness of the proposed method when applied to real fMRI data.

## 6. Conclusions

In this paper we proposed a variational Bayesian causal connectivity method for fMRI. The method uses a VAR model for the neuronal time series and the connectivity between regions in combination with a hemodynamic convolution model. By introducing an approximation to the neuronal time series and performing parts of the estimation in the frequency domain, our method is computationally efficient and can be applied to large scale problems with several hundred ROIs and high sampling rates.

We performed simulations with synthetic data to evaluate the performance of our method and to compare it with classical Wiener–Granger causality analysis (WGCA). There are several important findings from these simulations that need further discussion. In the first simulation, we demonstrated an important strength of our method, that is, it performs significantly better than WGCA when applied to problems with large numbers of regions. This effect is due to the use Gaussian priors for the VAR coefficients in combination with gamma priors for the precision hyperparameters. This prior has a regularizing effect by promoting sparsity for the VAR coefficients and can be seen as an adaptation of sparse Bayesian learning (Tipping, 2001) to the problem of VAR coefficient estimation. In contrast, WGCA does not use regularization for the VAR coefficients resulting in a performance degradation when the number of regions is increased. It is important to note that also the method in Ryali et al. (2011) employs Gaussian-gamma priors for the VAR coefficients. However, due to the computational complexity of the method it can only be applied to problems with small numbers of regions, where the prior is overwhelmed by the data and the sparsity promoting effect is of little benefit.

In the second set of simulations, we evaluated our method using simulated data generated by VAR processes of varying orders. Again, due to the prior for the VAR coefficients, where we group coefficients across lags together using shared precision hyperparameters, our method performed well as long as the VAR order used in the method is equal or higher than the VAR order of the data. A grouping of VAR coefficients using ℓ_1_ℓ_2_-norm regularization was first proposed in Haufe et al. (2008), in our work we propose a Bayesian formulation for this problem.

In the third simulation, we analyzed the effect of using an approximation to the neuronal time series, which is employed in our method to improve the computational efficiency, by comparing our method with a modified version of our method where the convolution with the HRF is included in the observation matrix of the linear dynamic system, as in previous methods (Smith et al., 2010; Ryali et al., 2011). The simulation results show that the approximation leads to some reduction in the quality of the estimated neuronal signal in terms of mean-squared error (MSE) but does not have a significant influence on the connectivity estimation performance. Importantly, the reduction in computational complexity resulting from the use of the approximation to the neuronal signal allows us to apply the method to large scale problems. As discussed above, the sparsity promoting priors for the VAR coefficients are of crucial importance when the method is applied to problems with large numbers of regions. The use of the approximation to the neuronal time series is therefore an important contribution of this work, as it allows us to apply the method to problem sizes where the method can benefit from the regularizing effect of the priors.

In a last set of simulations, we analyzed the effect of different downsampling ratios, simulating different TR values of the MRI scanner, the neuronal delay, and HRF variability. Perhaps not surprisingly, the proposed method is immune to HRF variability if it has access to the true HRF of each region. Clearly, in practice HRFs are subject and region dependent. However, it has been shown that HRFs are strongly correlated across subjects and regions (Handwerker et al., 2004). Hence, using data from a large number of subjects, it may be possible to construct a model describing the relationship between the HRFs in various brain regions. This “hemodynamic atlas” could then be used to approximate the HRFs in a large number of regions from a small number of estimated HRFs for each subject. We also found that the proposed method generally performs better than WGC when a significant amount of additive noise is present in the data. This finding is consistent with previous results (Seth et al., 2013) and can be explained by the model used in the proposed method which can account for additive noise. However, while the proposed method offers some benefits over WGC, we find that also the proposed method can estimate a causal influence with the opposite direction when the data has been downsampled, which is a known problem with WGC methods (David et al., 2008; Deshpande et al., 2010; Seth et al., 2013). The problem that causality estimated using a discrete-time VAR model from a sampled continuous-time VAR process can lead to opposite conclusions has been show before (Cox, 1992). Unfortunately, this problem has received little attention in recent work on causality estimation from fMRI data, where severe downsampling is common. In Solo (2007), it is shown that while causality can be preserved under downsampling, VAR models, as used in traditional WGC analysis and the proposed method, are inadequate for estimating causality from the subsampled time series and either VAR moving average (VARMA) models or state-space (SS) models are required to correctly estimate the direction of the causal influence. This raises hopes that causality estimation from fMRI may be feasible by applying more sophisticated models to data acquired with low TR values, which may be achieved using a combination of novel acquisition sequences and MRI scanners with higher field strengths. Clearly, HRF variability will still be a problem but under certain conditions it may be possible to use a model similar to the one proposed in this work which can take into account the HRF of each region.

Finally, we applied the proposed method to real resting-state fMRI data provided by the Human Connectome Project (Van Essen et al., 2012). For this data, the proposed method finds connections between regions that are associated with known resting-state networks. However, it is important to emphasize that application to real fMRI data as presented here serves as a demonstration to show that the proposed method can be applied to real fMRI data. As the true causal relationships in real data are not known, it not possible to determine whether the direction of causal influence is correctly estimated. As shown in our simulations, there are methodological problems which, depending on the noise level, the HRF, the TR, and the neuronal delay, can lead to causality inversions. Further experiments, e.g., using simultaneous EEG and fMRI, are necessary to quantify the effectiveness of the proposed method to estimate the direction of the causal influence from real fMRI data.

## Funding

This work was partially supported by the National Institute of Child Health and Human Development (R01 HD042049). Martin Luessi was partially supported by the Swiss National Science Foundation Early Postdoc Mobility fellowship 148485. This work was supported in part by the Department of Energy under Contract DE-NA0000457, the “Ministerio de Ciencia e Innovación” under Contract TIN2010-15137, and the CEI BioTic with the Universidad de Granada Data were provided (in part) by the Human Connectome Project, WU-Minn Consortium (Principal Investigators: David Van Essen and Kamil Ugurbil; 1U54MH091657) funded by the 16 NIH Institutes and Centers that support the NIH Blueprint for Neuroscience Research; and by the McDonnell Center for Systems Neuroscience at Washington University.

### Conflict of interest statement

The authors declare that the research was conducted in the absence of any commercial or financial relationships that could be construed as a potential conflict of interest.

## Footnotes

^1^Note that the only factorization we assume is the one in Equation (16); the induced factorizations appear in the derivation of the approximate posterior distribution and we can include them at this point to simplify the derivations.
